# Supplementary material for: Identification of photoperiod-induced specific miRNAs in the adrenal glands of Sunite sheep (Ovis aries)
Source: Front Vet Sci. 2022 Jul 22;9:888207. doi: 10.3389/fvets.2022.888207 (PMC9354845; doi:10.3389/fvets.2022.888207)
Supplement: Supplementary file 1 [file Data_Sheet_1.ZIP › Supplementary file/miRDeep2 file.zip!/miRDeep2 file/Know_Structure/SPLP42A3/oar-mir-29b-1.pdf]

miRBase precursor : oar-mir-29b-1
Total read count : 4452
oar-miR-29b read count : 4061
remaining reads : 391

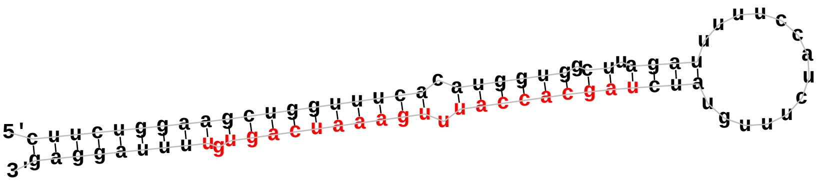


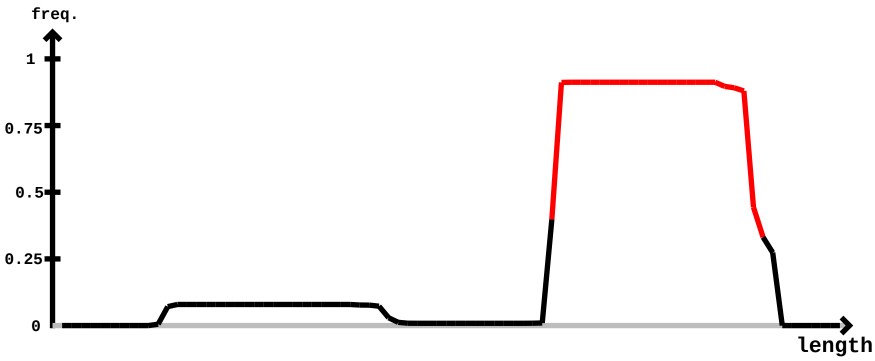


oar-miR-29b

```
5'- cuucuggaagcugguuucacaugguggcuuagauuuuuccaucuuuguaucuagcaccauuugaaaucaguguuuuaggag   -3'    exp
    (((((((((((((((((((.((((((.((.((((..............)))))))))))).)))))))))).)))))))))    reads      mm         sample
    .........gcugguuucacaugguggcuu...................................................    1          0          seq
    .........gcugguuucacaugguggcuua..................................................    1          0          seq
    .........gcugguuucacaugguggcuuag.................................................    1          0          seq
    .........gcugguuucacaugguggcuuaga................................................    20         0          seq
    ..........cugguuucacaugguggcuu...................................................    7          0          seq
    ..........cugguuucacaugguggcuua..................................................    1          0          seq
    ..........cugguuucacaugguggcuuag.................................................    16         0          seq
    ..........cugguuucacaugguggcuuaga................................................    192        0          seq
    ..........cugguuucacaugguggcuuagau...............................................    66         0          seq
    ..........cugguuucacaugguggcuuagauu..............................................    10         0          seq
    ..........cugguuucacaugguggcuuagauuu.............................................    1          0          seq
    ...........ugguuucacaugguggcuu...................................................    1          0          seq
    ...........ugguuucacaugguggcuuag.................................................    1          0          seq
    ...........ugguuucacaugguggcuuaga................................................    17         0          seq
    ...........ugguuucacaugguggcuuagau...............................................    12         0          seq
    ...........ugguuucacaugguggcuuagauu..............................................    5          0          seq
    .................................uuuuuccaucuuuguauA..............................    2          1          seq
    .................................uuCuuccaucuuuguauc..............................    1          1          seq
    .................................uuuuuccaucuuuguauU..............................    3          1          seq
    .................................uuuuuccaucuuuguauc..............................    29         0          seq
    .................................uuuuuccaucCuuguauc..............................    1          1          seq
    .................................uuuuuccaucuCuguauc..............................    1          1          seq
    ..................................uuuuccaucuuuguaucu.............................    1          0          seq
    ................................................aucuagcaccauuugaaaucagu..........    1          0          seq
    .................................................ucuagcaccauuugaaaucagu..........    4          0          seq
    ..................................................cuagcaccauuugaaauc.............    31         0          seq
    ..................................................cuaCcaccauuugaaauc.............    1          1          seq
    ..................................................cuagcaccauuugaaauca............    6          0          seq
    ..................................................cuagcaccauuugaaaucag...........    7          0          seq
    ..................................................cuagcaccauuugaaaucagu..........    1093       0          seq
    ..................................................cuagcaccauuugaaaucGgu..........    15         1          seq
    ..................................................cuagcaccauuugaaaucagug.........    387        0          seq
    ..................................................cuagcaccauuugaaaucagugu........    186        0          seq
    ..................................................cuagcaccauuugaaaucaguguu.......    3          0          seq
    ...................................................uagcaccauuugaaauU.............    2          1          seq
```

oar-miR-29b

```
cuucuggaagcugguuucacaugguggcuuagauuuuuccaucuuuguaucuagcaccauuugaaaucaguguuuuaggag
...................................................uagcaccauuugaaauc.............   33     0    seq
...................................................uagcaccauuugaaauA.............   2      1    seq
...................................................uagcaccauuugaaaucG............   10     1    seq
...................................................uagcaccauuugaaauca............   8      0    seq
...................................................uagcaccauuugaaaucag...........   13     0    seq
...................................................uagcaccauuugaaaucGg...........   32     1    seq
...................................................uagcaccauuugaaaucagu..........   586    0    seq
...................................................uagcaccauuugaaaucGgu..........   242    1    seq
...................................................uagcaccauuugaaaucagug.........   114    0    seq
...................................................uagcaccauuugaaaucagugu........   70     0    seq
...................................................uagcaccauuugaaaucaguguu.......   1212   0    seq
....................................................agcaccauuugaaaucaguguu.......   4      0    seq
```
